# Clinical Interventions During Inter‐hospital Transfer of Infants With Moderate‐to‐Severe Bronchiolitis: Implications for Advanced Nursing Practice

**DOI:** 10.1111/nicc.70460

**Published:** 2026-03-23

**Authors:** Arthur Gaudaire, Christophe Milesi, Alexia Morel, Julien Baleine, Maliha Badr, Alexandra Deveze, Sylvain Paulhac, Marion Palpacuer, Gilles Cambonie, Arthur Gavotto

**Affiliations:** ^1^ Department of Neonatal Medicine, Paediatric Intensive Care and Paediatric Emergency Transport Service, Arnaud de Villeneuve Hospital, Montpellier University Hospital Centre University of Montpellier Montpellier France; ^2^ PhyMedExp University of Montpellier, CNRS, INSERM Montpellier France; ^3^ Pathogenesis and Control of Chronic Infection, INSERM UMR 1058 University of Montpellier Montpellier France

**Keywords:** bronchiolitis, clinical decision‐making, inter‐hospital transport, nurse‐led team, Paediatric critical care nursing, respiratory support

## Abstract

**Background:**

Bronchiolitis is a leading cause of hospitalisation and paediatric intensive care admissions in infants. Moderate‐to‐severe cases often require inter‐hospital transfer for respiratory support, usually organised by specialised paediatric emergency transport services (PETS). The optimal composition of transport teams, whether nurse‐led or medicalised, remains uncertain.

**Aim:**

To identify predictive factors available during the regulation call that can help determine when an inter‐hospital transfer of infants with moderate‐to‐severe bronchiolitis can be safely conducted by a nurse‐led team without a paediatrician, by predicting the need for clinical intervention during transport.

**Study Design:**

A retrospective observational study was conducted from 2021 to 2023 within the PETS of a French University Hospital. Infants under 2 years transferred for moderate‐to‐severe bronchiolitis were included. The primary outcome was the occurrence of a clinical intervention during transport, defined as any event requiring physician‐level management: apnoea requiring manual ventilation, fluid bolus, initiation of two‐level non‐invasive ventilation or endotracheal intubation. Clinical and paraclinical parameters available at the regulation call—particularly ventilatory support mode, FiO_2_ and blood gas values—were analysed for their ability to predict the occurrence of such interventions using receiver operating characteristic (ROC) analysis.

**Results:**

Among 167 included infants (mean age 157 ± 169 days; weight 5.9 ± 2.7 kg), 20 (12%) required a clinical intervention. Higher FiO_2_ (51.3% ± 19.3% vs. 34.8% ± 10.2%; *p* < 0.01), lower pH (7.30 ± 0.08 vs. 7.34 ± 0.07; *p* = 0.03) and higher pCO_2_ (62.9 ± 17.9 vs. 49.6 ± 11.2 mmHg; *p* = 0.01) were associated with interventions. The presence of high‐flow nasal cannula (HFNC) with FiO_2_ > 40%, or continuous positive airway pressure (CPAP) with FiO_2_ > 35% or pCO_2_ > 65 mmHg predicted the need for a medicalised team (AUC = 0.83; sensitivity 90%, specificity 78%, negative predictive value 98%).

**Conclusions:**

Most inter‐hospital transfers of infants with moderate‐to‐severe bronchiolitis can be safely undertaken by nurse‐led teams when predefined respiratory or blood gas thresholds are not exceeded.

**Relevance to Clinical Practice:**

This study provides objective criteria to guide decision‐making regarding team composition during the regulation of inter‐hospital transfers for infants with moderate‐to‐severe bronchiolitis. FiO_2_ and pCO_2_ thresholds measured at the initial call can help identify cases requiring physician presence, while allowing most transfers to be safely conducted by nurse‐led teams. These results support the development of standardised triage protocols and strengthen the role of advanced paediatric critical care nurses in retrieval medicine. Integrating such evidence‐based criteria into practice could optimise human resources, maintain safety and improve response times in paediatric emergency transport systems.

## Impact Statements

- What is known about the topic
  ○ Bronchiolitis is a major cause of PICU transfer in infants.
  ○ Team composition during transport (nurse‐led vs. medicalised) remains debated.
  ○ Objective decision criteria are lacking in triage practice.
- What this paper adds
  ○ FiO_2_ > 40% (HFNC), or FiO_2_ > 35% (CPAP), or pCO_2_ > 65 mmHg predict need for medical team.
  ○ Most transfers can be safely managed by nurse‐led teams when below these thresholds.
  ○ Provides evidence‐based criteria to guide nursing triage and support advanced practice roles.

## 1 Introduction

Infant bronchiolitis is a contagious viral infection that affects approximately 480 000 children each winter in France, including nearly 30% of infants under 2 years of age. It accounts for 15%–17% of hospital admissions in this population [1, 2]. The main causative viruses are respiratory syncytial virus (RSV), responsible for about half of hospitalisations in infants under 6 months and rhinovirus [2]. First‐line treatment of bronchiolitis is symptomatic, consisting primarily of nasal suction. Oxygen dependency may occur, requiring oxygen therapy through a low‐flow system. In more severe cases, positive‐pressure respiratory support such as high‐flow nasal cannula (HFNC), continuous positive airway pressure (CPAP) or two‐level non‐invasive ventilation (NIV) may be needed [3, 4, 5, 6]. Intubation and mechanical ventilation are required in 4%–7% of patients admitted to paediatric intensive care units (PICUs) [5, 7].

Because these advanced respiratory techniques (HFNC, CPAP and NIV) are only available in PICUs, which are primarily located in tertiary or university hospitals, infants frequently require inter‐hospital transfer from non‐specialised facilities to a centre with intensive care capacity [8]. These secondary transfers are organised by specialised paediatric emergency transport services (PETS) [9].

Decisions regarding the composition of the transport team are made during a medical regulation call. Based on the infant's medical history, illness severity and the support required for vital functions, the regulating physician may dispatch either a nurse‐led inter‐hospital transfer, involving an advanced paediatric critical care nurse and an ambulance driver, or a medicalised transfer including a paediatrician. This decision has significant implications, as mobilising a full team temporarily reduces availability for primary emergency interventions, which remain the PETS' core mission.

The main objective of this study was to identify predictive criteria available during the regulation call to determine when an inter‐hospital transfer of infants with moderate‐to‐severe bronchiolitis can be safely performed by a nurse‐led team without a paediatrician.

## 2 Methods

### 2.1 Study Design and Population

This retrospective observational study was conducted within the Paediatric Emergency Transport Service (PETS) of Montpellier University Hospital, France, from January 2021 to December 2023. All infants under 2 years of age requiring inter‐hospital transfer for moderate‐to‐severe bronchiolitis were eligible. As no universally accepted definition of bronchiolitis severity exists, patient selection was based on the need for respiratory support (low‐flow oxygen, HFNC or CPAP).

Exclusion criteria were: (i) patients already intubated or receiving two‐level NIV at the time of the regulation call, (ii) patients breathing spontaneously without any ventilatory support at the time of the call, and (iii) patients with incomplete data. Infants breathing spontaneously (i.e., receiving no low‐flow oxygen, HFNC or CPAP) were excluded because they would not normally require PETS intervention, whereas intubated infants or those on two‐level NIV were excluded because our institutional protocol mandates paediatrician presence for their transport.

### 2.2 Regional Organisation of Paediatric Intensive Care and Moderate‐To‐Severe Bronchiolitis Management Protocol

When healthcare professionals request an inter‐hospital transfer, they contact the regional transfer regulation centre based at Montpellier University Hospital, which assigns a PETS team. The Montpellier PETS is the only service operating 24/7 in the eastern Occitanie region, where the only regional PICU is also located.

In France, the formal status of advanced paediatric or neonatal nurse is not officially recognised. However, in our institution, experienced nurses have developed advanced clinical and procedural expertise through structured in‐house training inspired by advanced nursing practice models from English‐speaking healthcare systems with formally recognised advanced nursing roles. These nurses are fully integrated into PETS activities and play a central role in both medicalised and nurse‐led inter‐hospital transfers.

Advanced paediatric critical care nurses working at PETS Montpellier are state‐registered paediatric nurses with postgraduate experience in neonatal and paediatric intensive care. Their scope of practice includes management of non‐invasive respiratory support (HFNC and CPAP), airway monitoring, peripheral venous access, gastric tube placement and administration of emergency treatments under predefined protocols. All are certified in Newborn Life Support (NLS) [10] and European Paediatric Advanced Life Support (EPALS) [11].

The core PETS team consists of a paediatrician and an advanced paediatric critical care nurse and is available 24 h a day, 7 days a week. In addition, a second senior nurse, also available 24/7 and based within the tertiary hospital, supports nurse‐led inter‐hospital transfers and provides delivery room assistance and participation in the in‐hospital mobile resuscitation team when required. During the study period, the mobile nursing team comprised 12 senior nurses and remained stable.

The management of infants with bronchiolitis during transfer follows a predefined service protocol based on ventilatory support. For moderate bronchiolitis (modified Wood's Clinical Asthma Score [mWCAS] [4] ≤ 4), transport is performed under low‐flow oxygen or high‐flow nasal cannula (HFNC). For severe bronchiolitis (mWCAS > 4), continuous positive airway pressure (CPAP) with a positive end‐expiratory pressure (PEEP) of 7 cmH_2_O is initiated, with escalation to two‐level NIV if the clinical condition worsens. Intubation is considered only as a last resort in cases of refractory hypoxia, recurrent apnoea or altered consciousness despite well‐administered NIV.

In France, while most emergency departments are able to initiate HFNC or CPAP in infants with bronchiolitis, the prolonged management of such non‐invasive respiratory support is usually limited to tertiary, university‐affiliated centres.

### 2.3 Data Collected

The following data were recorded: age at transfer, weight, identified virus, relevant comorbidities, capillary blood gas results (pH, pCO_2_ in mmHg) from the referring hospital, C‐reactive protein (CRP) levels (mg/L), chest X‐ray findings, mWCAS score and the presence of venous access or a nasogastric tube.

During the regulation call and throughout transport, ventilatory mode and FiO_2_ (%) were noted. The type of transfer decided by the regulating physician—nurse‐led or medicalised—was also documented.

### 2.4 Outcomes

The primary outcome was the occurrence of a clinical intervention during inter‐hospital transfer. This outcome was deliberately chosen as a pragmatic and clinically meaningful surrogate for situations requiring physician‐level management during transport. A clinical intervention was defined as any event requiring medical decision or advanced airway and haemodynamic management, including management of apnoea or respiratory distress requiring manual ventilation with a self‐inflating bag, fluid bolus administration, initiation of two‐level NIV, endotracheal intubation or cardiopulmonary resuscitation.

The secondary objective was to identify which clinical or paraclinical parameters available at the time of the regulation call could predict the occurrence of such interventions, in order to support decision‐making regarding team allocation (nurse‐led versus medicalised transfer).

### 2.5 Ethical and Institutional Approvals

The study complied with the principles of Good Clinical Practice and the Declaration of Helsinki. It was approved by the Institutional Review Board of Montpellier University Hospital (CSE‐2024‐06‐072) on July 24, 2024. In accordance with French legislation, written informed consent from participants or their legal guardians was not required.

### 2.6 Statistical Analysis

Comparisons were performed using Fisher's exact test, chi‐squared test, Student's *t*‐test or Wilcoxon–Mann–Whitney test, as appropriate. Predictive performance of the data provided during the regulation call was assessed using sensitivity, specificity, positive and negative predictive values and the area under the receiver operating characteristic (ROC) curve.

A *p*‐value < 0.05 was considered statistically significant. Analyses were performed using EasyMedStat version 3.21.5 (Paris, France).

## 3 Results

### 3.1 Study Population

Over the 3‐year study period, the PETS completed 2219 secondary transports. Among these, 190 (8.6%) involved infants with moderate‐to‐severe bronchiolitis. Forty‐three were excluded: 2 already intubated, 9 on two‐level NIV, 12 breathing spontaneously and 20 with incomplete data.

A total of 167 infants (77 boys, 90 girls) were included. The mean age was 157 ± 169 days, and mean weight was 5.9 ± 2.7 kg. Comorbidities included prematurity in 13% and congenital heart disease (CHD) in 8%. Viral identification was available in 66% of cases, among which RSV accounted for 81%. Table 1 summarises population characteristics by year.

**TABLE 1:** Population characteristics.

Variables		Total	2021	2022	2023
*N*		167	47	71	49
Patient's characteristics					
Male (yes)		77 (46)	23 (49)	28 (39)	26 (53)
Age (days)		157 ± 169	137 ± 145	138 ± 168	202 ± 184
Weight (Kg)		5.9 ± 2.7	5.7 ± 2.3	5.4 ± 2.5	6.7 ± 2.9
Virus (*N* = 111)	RSV	90 (81)	34 (87)	37 (79)	19 (76)
	Others	21 (19)	5 (13)	10 (21)	6 (24)
Significant history	Prematurity	21 (13)	3 (6)	10 (14)	8 (16)
	CHD	13 (8)	3 (6)	6 (9)	4 (8)
Description of regulation call					
Chest X‐ray (*N* = 97)	Atelectasis	19 (20)	3 (10)	12 (34)	4 (12)
	Pneumopathy	18 (19)	4 (14)	5 (14)	9 (27)
mWCAS (*N* = 47)		4.8 ± 1.7	4.2 ± 1.2	5.0 ± 2.0	5.2 ± 1.7
Blood gas (*N* = 95)	pH	7.33 ± 0.07	7.35 ± 0.06	7.34 ± 0.08	7.31 ± 0.08
	pCO_2_ (mmHg)	52.1 ± 13.5	50.0 ± 10.4	53.0 ± 15.9	53.4 ± 13.1
CRP (mg/L) (*N* = 103)		33.1 ± 49.8	34.0 ± 42.5	26.4 ± 40.2	39.4 ± 63.5
Venous access (yes)		112 (67)	36 (77)	42 (59)	35 (71)
Nasogastric tube (yes)		73 (44)	20 (43)	32 (45)	21 (43)
Ventilatory support	Low‐flow O_2_	29 (17)	6 (13)	15 (21)	8 (16)
	HFNC	88 (53)	28 (59)	34 (48)	26 (53)
	CPAP	50 (30)	13 (28)	22 (31)	15 (31)
FiO_2_ (%)		37.2 ± 13.1	38.3 ± 11.4	38.7 ± 15.6	33.9 ± 10.1
Paediatric emergency medical service					
Full team (yes)		158 (95)	46 (98)	66 (93)	46 (94)
Upgrade in respiratory support (yes)		47 (28)	19 (40)	15 (21)	13 (26)
Upgrade to NIV or intubation (yes)		15 (9)	7 (15)	2 (3)	6 (12)
Ventilatory support	Low‐flow O_2_	23 (14)	5 (11)	11 (15)	7 (14)
	HFNC	58 (35)	11 (23)	27 (38)	20 (41)
	CPAP	70 (42)	25 (53)	31 (44)	14 (29)
	NIV	16 (10)	6 (13)	2 (3)	8 (16)

*Note:* Values are expressed as mean ± standard deviation or *n* (%).

Abbreviations: CHD, congenital heart defect; CPAP, continuous positive airway pressure; HFNC, high‐flow nasal cannula; mWCAS, modified Wood's Clinical Asthma Score; NIV, non‐invasive ventilation; RSV, respiratory syncytial virus.

### 3.2 Data Available at the Regulation Call

At the time of the regulation call, 88 infants (53%) were receiving HFNC (mean FiO_2_ 36.8% ± 13.0%), 50 (30%) were on CPAP (mean FiO_2_ 37.8% ± 13.6%) and 29 (17%) were on low‐flow oxygen.

The mWCAS score was reported in 28% of cases (mean 4.8 ± 1.7). Chest X‐rays were performed in 97 infants (58%), revealing atelectasis in 20% and pneumonia in 19% of cases. A capillary blood gas was available in 95 cases (57%), and CRP levels in 103 (62%). Venous access was present in 67% of infants, and 44% had a nasogastric tube (Table 1).

### 3.3 Clinical Intervention During Transport

A complete team (paediatrician, advanced paediatric critical care nurse and driver) was dispatched in 158 transfers (95%).

A clinical intervention occurred in 20 cases (12%), including 15 NIV initiations, 4 manual ventilations for apnoea and 1 previously undiagnosed cyanogenic CHD.

An additional 32 infants experienced escalation of respiratory support (25 from HFNC to CPAP, and 7 from low‐flow oxygen to HFNC), which were managed by nurse‐led teams according to protocol (Table 1).

### 3.4 Prediction of Clinical Intervention

Table 2 compares characteristics at the regulation call according to the occurrence of an intervention requiring a complete team. Infants who required intervention had higher FiO_2_ (51.3% ± 19.3% vs. 34.8% ± 10.2%; *p* < 0.01), lower pH (7.30 ± 0.08 vs. 7.34 ± 0.07; *p* = 0.03) and higher pCO_2_ (62.9 ± 17.9 vs. 49.6 ± 11.2 mmHg; *p* = 0.01). Other collected variables (e.g., age, comorbidities, CRP level, chest X‐ray findings and viral identification) were not significantly associated with clinical intervention.

**TABLE 2:** Comparison between simple transport and transport needing clinical intervention.

Variables		Simple transport (*N* = 147)	Medical intervention (*N* = 20)	*p*
Male (yes)		68 (46)	9 (45)	0.99
Age (days)		162 ± 176	116 ± 108	0.51
Weight (Kg)		5.9 ± 2.7	5.2 ± 2.4	0.33
RSV (yes)		78/97 (81)	12/14 (86)	0.40
Description of regulation call				
Chest X‐ray (*N* = 97)	Atelectasis	17/83 (20)	2/14 (14)	0.10
	Pneumopathy	18/83 (22)	0/14 (0)	
Blood gas (*N* = 95)	pH	7.34 ± 0.07	7.30 ± 0.08	0.03
	pCO_2_ (mmHg)	49.6 ± 11.2	62.9 ± 17.9	0.01
CRP (mg/L) (*N* = 103)		35.4 ± 51.1	18.5 ± 41.4	0.11
mWCAS (*N* = 47)		4.6 ± 1.8	5.6 ± 0.8	0.06
Ventilatory support	Low‐flow O_2_	29 (20)	0 (0)	0.09
	HFNC	75 (51)	13 (65)	
	CPAP	43 (29)	7 (35)	
FiO_2_ (%)		34.8 ± 10.2	51.3 ± 19.3	< 0.01

*Note:* Values are expressed as mean ± standard deviation or *n* (%).

Abbreviations: CPAP, continuous positive airway pressure; HFNC, high‐flow nasal cannula; mWCAS, modified Wood's Clinical Asthma Score; NIV, non‐invasive ventilation; RSV, respiratory syncytial virus.

ROC analyses showed that: (i) CPAP with FiO_2_ > 35% predicted the need for a medicalised team (AUC = 0.83; sensitivity 86%; specificity 63%), (ii) HFNC with FiO_2_ > 40% also predicted clinical intervention (AUC = 0.78; sensitivity 85%; specificity 73%), and (iii) pCO_2_ > 65 mmHg was a strong indicator (AUC = 0.73; sensitivity 56%; specificity 94%). Because mWCAS was available in only 28% of cases and was frequently missing at the time of the regulation call, it was not suitable for reliable ROC analysis.

**FIGURE 1:**
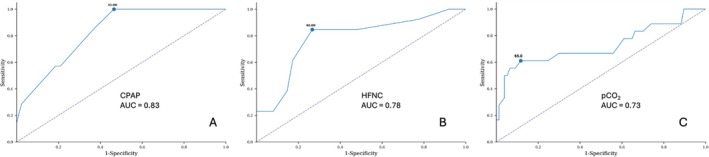
ROC curves. ROC curves predicting the occurrence of clinical intervention (A) CPAP FiO_2_, (B) HFNC FiO_2_ and (C) pCO_2_. AUC, area under the curve; CPAP, continuous positive airway pressure; HFNC, high‐flow nasal cannula.

Combining these three criteria at the regulation call (HFNC FiO_2_ > 40%, or CPAP FiO_2_ > 35%, or pCO_2_ > 65 mmHg) yielded a sensitivity of 90%, specificity of 78% and negative predictive value of 98% for predicting clinical intervention (Figure 1).

## 4 Discussion

This 3‐year retrospective observational study included 167 infants under 2 years of age transferred for moderate‐to‐severe bronchiolitis. A complete medical team was mobilised in 95% of transports, yet clinical interventions requiring physician presence occurred in only 12% of cases. Most of these situations were predictable from parameters available at the regulation call, particularly HFNC with FiO_2_ > 40%, or CPAP with FiO_2_ > 35%, or pCO_2_ > 65 mmHg. Applying these thresholds would have allowed 70% of transfers to be safely managed by nurse‐led teams.

These findings confirm that simple, objective respiratory criteria can reliably guide the choice of transport team. Similar observations have been reported internationally, suggesting that optimising triage for medical versus nurse‐led retrieval could reduce unnecessary mobilisation of paediatricians while maintaining safety and efficiency [12]. Although the negative predictive value of this predictive model was not 100%, the few discordant cases reflected physician discretion rather than protocol failure. The indication for NIV remains variable among physicians, despite the lack of evidence that NIV offers advantages over CPAP during transport [13].

This study was designed with a pragmatic, real‐life perspective, reflecting the conditions under which triage decisions are actually made during paediatric emergency transport regulation. Rather than relying on idealised severity classifications or systematically recorded clinical scores, we chose to focus on objective parameters that are consistently available at the time of the regulation call, such as ventilatory support modality, FiO_2_ and blood gas values. This approach aims to enhance the clinical applicability of our findings and to support decision‐making in everyday practice.

The high proportion of infants initially supported with HFNC or CPAP by referring hospitals demonstrates the success of regional training and standardised care protocols [14]. These initiatives have enhanced local stabilisation capacity and decreased the need for invasive ventilation before transport. Most infants also had venous access and a nasogastric tube inserted before arrival of the transport team, highlighting improved anticipation by bedside nurses. Nasogastric decompression optimises positive‐pressure ventilation and enables enteral feeding, which has been associated with shorter hospital stays and lower ventilatory requirements [15, 16, 17, 18].

Our data suggest that, in most cases, the inter‐hospital transfer of infants with bronchiolitis can be safely performed by advanced paediatric critical care nurses. Within our PETS, these nurses are experienced in intensive and emergency care, certified in both NLS and EPALS and trained specifically for paediatric transport [10, 11]. Their role includes ensuring airway stability, adjusting FiO_2_, performing procedures such as venous or gastric tube placement, and administering treatments under pre‐established protocols. These findings are consistent with recent international literature describing the expanding role of advanced nursing practitioners in paediatric critical care transport services. In particular, Herring et al. highlighted the contribution of advanced clinical practitioners to paediatric retrieval teams, supporting the feasibility and safety of nurse‐led transport models within well‐defined governance frameworks [19]. It is important to note that the scope of practice of advanced paediatric critical care nurses in France differs from that of advanced nurse practitioners described in other healthcare systems, particularly in English‐speaking healthcare systems where such roles are formally recognised. French transport nurses do not perform endotracheal intubation or prescribe independently. Expanding the scope of nurse‐led transfers could therefore optimise resource allocation, maintain paediatrician availability for primary emergencies and reduce operational costs while preserving patient safety.

### 4.1 Study Limitations

This study has several limitations. Its retrospective design led to missing data, notably regarding the mWCAS, which plays a key role in assessing severity and guiding ventilatory management. The score was documented in only 28% of cases, particularly infrequently at the time of the regulation call, even though part of the clinical decision‐making relies on it. Because of this inconsistent documentation in routine practice, we deliberately adopted a pragmatic approach, prioritising clinical and paraclinical parameters that are routinely and reliably available at the time of the regulation call over formal severity scores. However, this limitation could be easily overcome in daily practice, as such clinical scoring is feasible even through videoconferencing.

The definition of ‘clinical intervention’ was based on pragmatic criteria and could be debated; some upgrades in respiratory support (low‐flow oxygen to HFNC or HFNC to CPAP) were not classified as interventions, as they are permitted within nursing competence under current protocols.

As a single‐centre study, our results may not be directly generalisable to other regions where paediatric emergency transport and nursing expertise differ. However, our findings are consistent with previous research from our team on bronchiolitis management and respiratory support in infants, which supports their internal validity and contextual relevance [3, 4, 5, 6]. This coherence supports the internal validity of our results and reinforces their potential applicability within similar organisational models.

Data on race or ethnicity were not available in this retrospective cohort, as their collection is strictly regulated by law in France and rarely included in routine clinical datasets outside specific ethically approved research frameworks.

Finally, data were collected between 2021 and 2023, a period marked by the major 2022 RSV epidemic and the subsequent introduction of preventive strategies such as maternal vaccination and monoclonal antibody prophylaxis [20, 21]. Ongoing surveillance is warranted to confirm whether these evolving epidemiological trends affect future transport needs and team composition.

### 4.2 Implications for Practice

This study highlights the potential for advanced paediatric critical care nurses to safely conduct most inter‐hospital transfers of infants with moderate‐to‐severe bronchiolitis. Objective respiratory thresholds, particularly HFNC with FiO_2_ > 40% or CPAP with FiO_2_ > 35% or pCO_2_ > 65 mmHg, can guide team allocation during the regulation call, helping to identify which situations require a medicalised team. Implementing these evidence‐based criteria within triage protocols could enhance nurses' clinical autonomy, standardise decision‐making and improve the efficiency of emergency transport systems.

Expanding nurse‐led transfers may also optimise resource utilisation by preserving paediatricians for primary emergencies while maintaining patient safety. Ongoing training in ventilatory management, simulation exercises and strong interprofessional collaboration remain essential to consolidate these advanced competencies and ensure consistent, high‐quality care during paediatric retrieval.

## 5 Conclusion

Most inter‐hospital transfers of infants with moderate‐to‐severe bronchiolitis can be safely conducted by nurse‐led teams when predefined respiratory and blood gas thresholds are not exceeded. FiO_2_ and pCO_2_ values available at the regulation call provide simple and reliable indicators to guide team allocation. These findings support the development of standardised, evidence‐based triage protocols to strengthen nurses' autonomy and optimise resource use in paediatric emergency transport.

Future multicentre studies should assess the safety and cost‐effectiveness of this nurse‐led model in different organisational contexts and explore its integration into advanced nursing education, competency assessment and practice frameworks.

## Author Contributions

A.G., A.G., C.M. and PETS' advanced paediatric critical care nurses contributed to the study conception and design. Analysis was performed by A.G. Material preparation and data collection were performed by all authors. The first draft of the manuscript was written by A.G., A.G., and all authors commented on previous versions of the manuscript. All authors read and approved the final manuscript.

## Funding

This work was supported by the Montpellier University Hospital, France.

## Ethics Statement

Approved by the Institutional Review Board of Montpellier University Hospital (CSE‐2024‐06‐072, approval date: June 2024).

## Consent

In accordance with French legislation, written informed consent from participants or their legal guardians was not required.

## Conflicts of Interest

The authors declare no conflicts of interest.

## Data Availability

The data that support the findings of this study are available on request from the corresponding author. The data are not publicly available due to privacy or ethical restrictions.
